# Is a Mean Machine Better than a Dependable Drive? It’s Geared Toward Your Regulatory Focus

**DOI:** 10.3389/fpsyg.2012.00268

**Published:** 2012-08-07

**Authors:** Graham G. Scott, Sara C. Sereno, Patrick J. O’Donnell

**Affiliations:** ^1^Department of Psychology, University of BedfordshireLuton, UK; ^2^Institute of Neuroscience and Psychology, University of GlasgowGlasgow, UK; ^3^School of Psychology, University of GlasgowGlasgow, UK

**Keywords:** emotion, words, messages, regulatory focus, attitude change

## Abstract

While many studies have investigated the role of message-level valence in persuasive messages (i.e., how positive or negative message content affects attitudes), none of these have examined whether word-level valence can modulate such effects. We investigated whether emotional language used within persuasive messages influenced attitudes and whether the processing of such communications could be modulated by regulatory focus. Using a 2 (Message: Positive, Negative) × 2 (Words: Positive, Negative) design, participants read car reviews and rated each on a series of semantic differentials and product recommendations. While positive messages were always rated higher than negative ones, the valence of a message’s component words differentially impacted attitudes toward distinct aspects of the product. On promotion-focus features, messages containing negative words produced higher ratings; for prevention-focus aspects, those with positive words resulted in higher ratings. We argue that adopting a prevention- or promotion-focused stance can influence the interpretation of emotion words in relation to overall message comprehension.

## Introduction

Text has long been used in order to shape the attitudes of individuals. The role of positive and negative information as drivers of attitude change has been well-explored in the persuasion literature. Topics extensively investigated include the impact of framing, the presence of regulatory focus variables such as the promotion- or prevention-focus of a message’s arguments, and the role of two-sided communications (Cesario et al., 2008; Rucker et al., 2008). The focus in such inquiries has always been on the valence of information presented (e.g., an overall positive appraisal with some minor negative information included) rather than the composition of the message. While it is well-established that advertisers woo in part through emotionally manipulating their audience, no study to our knowledge has investigated the relative contribution of component emotion words to convey persuasive messages and to determine their role in attitude formation.

The overwhelming focus of persuasion research has been on message-level features, with less interest in lower-level aspects of a message, such as the use of emotional language. Even studies which use processing measures such as speed of comprehension are relatively rare. This lack of interest in the psycholinguistic properties of the message is curious given the weight of research which has investigated the interaction of the semantic properties of words with discourse-level effects (e.g., Morris, 1994; Garrod and Terras, 2000; Hagoort et al., 2004; Sereno et al., 2006). A message can be conceptualized at the discourse-level, where a general narrative and scenario context are established. It can also be conceptualized at the level of individual words which, through their own semantic properties, can themselves establish scenarios and causal relationships, and can create an emotional environment. There is a continuous interaction between the two levels in determining the overall communicative capacity of a message.

It is therefore necessary to first consider how we process emotional (positive and negative) words. Advances have been made over the past two decades in understanding how emotion words are recognized – for example, a word’s valence has been shown to interact with word frequency (i.e., how often that word occurs in the language), thus demonstrating that emotional tone affects the earliest stages of processing (Kuchinke et al., 2007; Scott et al., 2009). Such effects have recently been found within the context of a natural reading situation (Scott et al., 2012), fueling suggestions they could interact with discourse effects, influencing higher-level processes. To our knowledge, however, emotion words have never been manipulated independently of overall message valence in an attempt to influence attitudes.

An unresolved issue in emotion word recognition concerns to what extent emotional valence persists and is, consequently, able to influence higher-level cognitive functions. Suggestive evidence comes from research using emotional pictures which has demonstrated electrophysiological (EEG) effects lasting up to 6 s after stimulus presentation (e.g., Pastor et al., 2008). However, the impact of such pictures was not examined in relation to an overall “message.” It is difficult to apply our knowledge of how we process single emotion words (in isolation or in a sentence) to whether a persuasive message utilizing numerous emotion words can influence attitude formation. Additionally, whether message- and word-level valences are consistent (e.g., positive–positive or negative–negative) or not (e.g., positive–negative or negative–positive) may complicate the interpretation of such results.

A final topic related to the interpretation of persuasive messages is that of regulatory focus, in which an individual’s goal orientation influences their style of processing. According to Higgins (1998), a judgment outcome depends primarily on whether that person is promotion-focused (maximizing their gains) or prevention-focused (minimizing their losses). Regulatory focus theory (Higgins, 1998) argues that, in the pursuit of goals, individuals differ in how they self-regulate in their cognitive and behavioral strategies. Individuals with a promotion-focus work to achieve their ideal self, focus on the presence or absence of positive outcomes, and pursue their goals with eager anticipation, often prepared to take risks. Prevention-focused individuals, in contrast, are preoccupied with the person they ought to be, concentrate on the presence or absence of negative outcomes, and pursue their goals in a cautious, risk-adverse manner. As such, regulatory focus is orthogonal to reward-penalty outcomes. Identical outcomes, however, are achieved via different methods of operating (e.g., seeking hits vs. avoiding misses).

Regulatory focus exists as a chronic disposition, but it can also be induced or primed as a temporary orientation. Because regulatory focus, either as a state or a trait, is an individual difference, it operates as an additional factor in decision making. For example, in prospect theory, decision making is driven more by outcomes construed as certain as opposed to probable (Kahneman and Tversky, 1984). Accordingly, the relative weightings attached to positive and negative prospects will vary as a function of an individual’s regulatory focus (e.g., Kluger et al., 2004; Halamish et al., 2008). Regulatory focus theory has also been applied to decision making in consumer choice (e.g., Florack et al., 2010; Trudel et al., 2012). Moreover, regulatory focus has been shown to affect behavior across a number of contexts. For example, promotion-focus leads to earlier onset of goal pursuit (Freitas and Higgins, 2002), and makes one more likely to sit closer to an in-group member (Shah et al., 2004). Finally, an individual’s regulatory focus has been shown to elicit asymmetric frontal cortical brain activity thought to be associated with underlying motivational and emotional processing (Amodio et al., 2004).

The role of regulatory focus in message analysis is unclear. Pham and Avnet (2004) showed that, when primed with promotion goals, participants attend to the affect elicited by the persuasive message, while those primed with prevention goals attend to the content of that message. In a related consumer study, however, Zhang et al. (2010) showed that the persuasiveness of on-line product reviews was modulated by an interaction between the regulatory focus appeal of the product and message valence, with promotion-focused individuals showing a positivity bias and prevention-focused individuals a negativity bias. That study, however, was entirely between-participants. Chatterjee et al. (2010) found that regulatory focus interacted with mixed- versus blocked-sequencing of promotion and prevention features in brand evaluation. Specifically, they demonstrated that alternating promotion and prevention features improved brand evaluation among promotion- but not prevention-focused consumers.

The issue of regulatory fit has also been shown to be relevant to persuasion. Regulatory fit, defined as a match between regulatory focus of the individual and the promotion- or prevention-appeal of the message, leads to greater ease of message processing and a transfer of value to the target (Freitas and Higgins, 2002; Cesario et al., 2008). In consumer decision making, Mourali and Pons (2009) found that attribute-based, as opposed to alternative-based, processing showed a better fit with prevention-focused individuals leading to higher product valuation.

The interaction between regulatory focus and message and word valence effects is, however, an open question. The valence of a message or word *per se* is technically orthogonal to its regulatory focus which is couched in terms of gains versus losses. That is, the valence dimension of positive–negative does not correspond to the regulatory focus dimension of promotion-prevention. At the level of individual words used in many persuasive messages, however, it is possible that these two dimensions are correlated. Accordingly, it is possible that the use of emotionally valenced words, independent of message valence, will selectively modulate judgments related to promotion- and prevention-focused perceptions.

The current experiment sought to identify the message- and word-level factors which influence consumer choice in written advertisements. Message- and word-level valence of automobile reviews were orthogonally manipulated, giving rise to four message-word (M-W) conditions, each represented by a different review (i.e., positive–positive, positive–negative, negative–positive, and negative–negative). Participants read each review and their reading times were measured. After each review, participants responded to a series of semantic differentials that surveyed their attitudes across seven dimensions, three of which were promotion-oriented (i.e., speed, excitement, and fun), three of which were prevention-oriented (i.e., efficiency, comfort, and safety), and one of which was neither (desirability). Finally, they judged the suitability of each car for two potential buyers having attributes indicative of either promotion or prevention regulatory focus. These manipulations of regulatory focus are indirect. That is, we are not contrasting the behavior of promotion- and prevention-focused groups. Nevertheless, having participants respond to promotion- or prevention-oriented semantic differentials and requiring them to consider in their recommendations the mindset of promotion- and prevention-focused consumers, does induce them to momentarily adopt a promotion- or prevention-oriented stance.

## Materials and Methods

### Participants

Forty-eight members of the University of Glasgow community (33 female; mean age 21) received £4 or course credit for their participation. All had normal or corrected-to-normal vision, were right-handed, were native English speakers who had not been diagnosed as dyslexic, and were naïve as to the purpose of the experiment.

### Apparatus

Car reviews were presented on a Mac G4 (OS 9.0.4) computer using PsyScope 1.2.5 PPC software. The text was presented in 26-point Times New Roman font (black characters on a white background) on a Hansol 2100A 19″color monitor (100 Hz, 1024 × 768 resolution). At a viewing distance of approximately 86 cm, three characters on average subtended 1° of visual angle. Responses were made via a PsyScope Button Box and responses were recorded with millisecond accuracy.

Two questionnaires were administered following each car review. First, for each product, there was a set of seven 7-point semantic differentials. Three of these were related to a promotion-focus of eagerness and approach (Fast-Slow, Exciting-Calm, and Fun-Serious), three were related to a prevent focus of protection and security (Efficient-Wasteful, Comfortable-Uncomfortable, and Safe-Dangerous), and one was an overall product rating (Desirable-Undesirable). Second, there was a set of two 7-point product recommendations – for a promotion- and a prevention-focused individual.

To confirm the promotion or prevention bias of the six semantic differentials, each was rated on a 7-point Likert scale by seven additional participants. Fast-Slow, Exciting-Calm, and Fun-Serious were significantly rated as more promotion-focused [*t*(6) = 3.33, *p* < 0.05, *t*(6) = 2.57, *p* < 0.05, and *t*(6) = 8.00, *p* < 0.001, respectively]. Efficient-Wasteful and Safe-Dangerous were significantly rated as more prevention-focused [*t*(6) = 4.77, *p* < 0.01, *t*(6) = 8.22, *p* < 0.001], while Comfortable-Uncomfortable was rated marginally so [*t*(6) = 1.99, *p* = 0.094].

### Materials and design

A 2 (Message: Positive, Negative) × 2 (Word: Positive, Negative) within-participants design was used. One car review was created for the resulting four M-W conditions: Positive–Positive (+M+W, e.g., “*pristine handling and phenomenal grip which ensures supreme confidence when cornering*”); Positive–Negative (+M−W, e.g., “*ferocious speed, with razor-sharp handling allowing you to attack the tightest corners with venom*”); Negative–Positive (−M+W, e.g., “*new suspension offering the poise and grace of rollercoaster cart*”); and Negative–Negative (−M−W, e.g., “*the worst bumps can be felt as bone-shaking shudders*”). The four car ads – for the Ford Kiss (+M+W), Daewoo Scorpion (+M−W), Fiat Casino (−M+W), and Nissan Storm (−M−W) – appear in full in the Appendix. Each review comprised three paragraphs – the first on the car’s performance, the second on its comfort and size, and the third on its safety and efficiency. The emotion words used in the ads were rated by two independent groups of 10 participants. One group rated the words on valence using a scale of 1 (negative) to 7 (positive), and the other rated the words on arousal using a scale of 1 (low arousal) to 7 (high arousal). The text specifications for each car ad, including the number of emotion words contained in each paragraph and their emotional rating, are summarized in Table 1. The order of presentation of the four car reviews was randomized across participants according to a Latin-square design.

**Table 1:** **Text characteristics of car reviews**.

Paragraph	Message-Word condition	Lines	Words	Chars	Emotion words		
					*N*	Arousal	Valence
1	Positive–Positive	8	99	617	19	4.22	5.41
	Positive–Negative	8	114	675	18	4.12	2.33
	Negative–Positive	8	110	608	19	3.76	5.17
	Negative–Negative	8	99	625	19	3.98	2.14
2	Positive–Positive	4	50	321	9	3.97	5.37
	Positive–Negative	5	68	389	9	3.77	2.58
	Negative–Positive	4	47	278	10	3.80	5.41
	Negative–Negative	5	55	373	12	3.17	2.55
3	Positive–Positive	5	56	356	11	3.58	5.42
	Positive–Negative	5	52	335	12	3.68	1.88
	Negative–Positive	5	62	337	10	3.56	5.19
	Negative–Negative	4	49	312	18	3.99	2.06
Total	Positive–Positive	17	205	1294	39	3.92	5.40
	Positive–Negative	18	234	1399	39	3.85	2.25
	Negative–Positive	17	219	1223	39	3.70	5.24
	Negative–Negative	17	203	1310	39	3.71	2.21

*Rating scales for emotion words: Arousal, from 1 (low) to 7 (high), and Valence, from 1 (negative) to 7 (positive); Chars, characters*.

The first six 7-point semantic differentials (Fast-Slow, Exciting-Calm, Fun-Serious, Efficient-Wasteful, Comfortable-Uncomfortable, and Safe-Dangerous) appeared in one of two random orders, and Desirable-Undesirable was always presented last. Scale endpoints were reversed in different versions (e.g., 1 = Slow and 7 = Fast, or 1 = Fast and 7 = Slow). The recommendation questionnaire consisted of descriptions of two individuals (in random order) each of which induced a regulatory focus: Greg, a young executive concerned with wealth and status (promotion-focus), and Jane, a single parent worried about safety and economy (prevention-focus). Each description was followed by a 7-point scale to indicate a recommendation, with endpoints of “Would not recommend” (1) and “Would highly recommend” (7).

### Procedure

Participants were given informed consent and task instructions. Each product review was read and responded to in full before the next one was presented. Participants first read each review in a self-paced reading format, pressing a key when they had finished reading each of the three component paragraphs of that review. They then responded to the seven semantic differentials concerning the review just read. Finally, they provided their recommendations.

## Results

A 2 (Message: Positive, Negative) × 2 (Words: Positive, Negative) analysis of variance (ANOVA) was performed on the following measures: (1) car ad reading times; (2) each of the seven semantic differentials; and (3) the promotion- and prevention-focused recommendations.

### Reading time

**Figure 1:**
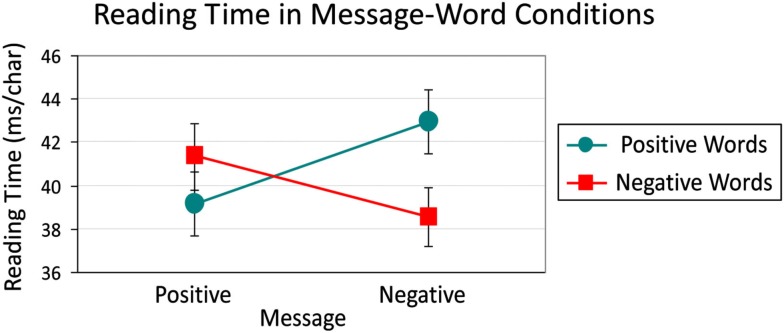
**Reading time (ms/char) with standard error bars across Message × Word conditions**.

For each car ad, the reading times for the component three paragraphs were summed and divided by the total number of characters (see Table 1) to obtain a millisecond-per-character (ms/char) measure. Condition means (with standard error bars) are presented in Figure 1. Neither of the main effects were significant [Message: *F* < 1; Word: *F*(1, 47) = 2.30, *MSE*  = 25, *p* = 0.136]. The Message × Word interaction, however, was significant [*F*(1, 47) = 23.97, *MSE*  = 22, *p* < 0.001]. Follow-up contrasts revealed significant effects of M-W consistency. That is, Positive Messages containing Positive Words (39.14 ms/char) were read faster than Positive Messages that contained Negative Words (41.35 ms/char; *F* = 5.32, *p* < 0.05). Similarly, Negative Messages with Negative Words (38.50 ms/char) were read faster than Negative Messages containing Positive Words (42.91 ms/char; *F* = 21.32, *p* < 0.001). Additionally, when the text used Positive Words, reading time was shorter when these words were embedded in a Positive versus a Negative Message (39.14 versus 42.91 ms/char; *F* = 15.58, *p* < 0.001). Likewise, when the text contained Negative Words, reading time was shorter when these words were embedded in a Negative versus a Positive Message (38.50 versus 41.35 ms/char; *F* = 8.86, *p* < 0.01).

### Semantic differentials

**Figure 2:**
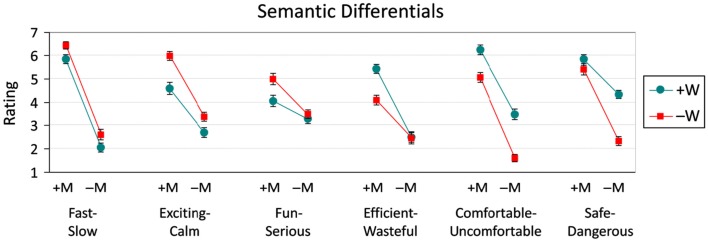
**Semantic differential ratings (with standard error bars) across Message (M) × Word (W) conditions**. M and W conditions were either Positive (+) or Negative (−). Promotion-oriented differentials include Fast-Slow, Exciting-Calm, and Fun-Serious. Prevention-oriented differentials include Efficient-Wasteful, Comfortable-Uncomfortable, and Safe-Dangerous. Each rating scale is 1 to 7, with 7 representing the first word of each pair.

Each semantic differential is examined separately below and the mean ratings across all differentials (with standard error bars) are presented in Figure 2. In general, cars described in Positive messages led to higher ratings than those described in Negative messages, regardless of the type of emotion words that were used to convey that message. Within Positive and Negative messages, however, the pattern of ratings was modulated by whether Positive or Negative words were utilized. Additionally, these effects were dependent on the nature of the semantic differential – that is, whether it was related to promotion- or prevention-focused aspects of the product.

#### Fast-Slow

There were significant main effects of both Message [*F*(1, 47) = 259.64, *MSE*  = 2.7, *p* < 0.001] and Word [*F*(1, 47) = 10.15, *MSE*  = 1.6, *p* < 0.01], but the interaction was not significant (*F* < 1). Positive messages led to higher ratings – cars described in Positive messages (6.14) were rated as being faster than those in Negative ones (2.34). However, car ads that used Negative words (4.53) were rated as being faster than those using Positive words (3.95).

#### Exciting-Calm

There were significant main effects of both Message [*F*(1, 47) = 98.83, *MSE*  = 2.4, *p* < 0.001] and Word [*F*(1, 47) = 24.19, *MSE*  = 2.1, *p* < 0.001], but the interaction did not reach significance [*F*(1, 47) = 2.40, *MSE*  = 2.7, *p* = 0.128]. The pattern of effects was similar to that for Fast-Slow. Positive messages (5.28) were rated as more exciting than Negative ones (3.04). Also, the use of Negative words (4.68) gave rise to higher ratings of excitement than did the use of Positive words (3.65).

#### Fun-Serious

In addition to significant main effects of Message [*F*(1, 47) = 15.32, *MSE*  = 4.0, *p* < 0.001] and Word [*F*(1, 47) = 6.33, *MSE*  = 2.5, *p* < 0.05], the interaction was also significant [*F*(1, 47) = 4.62, *MSE*  = 1.5, *p* < 0.05]. For the main effects, as with Fast-Slow, Positive messages (4.52) were rated higher in terms of fun than Negative ones (3.39), and messages containing Negative words (4.24) were rated as more fun than those with Positive words (3.67).

Follow-up contrasts to the interaction were significant with one exception detailed below. In line with the main effect of Message, Positive messages were rated as more fun than corresponding Negative ones: +M+W (4.04) was higher than −M+W (3.29; *F* = 8.74, *p* < 0.01), and +M−W (5.00) was higher than −M−W (3.48; *F* = 35.93, *p* < 0.001). With respect to effects due to the use of emotion words, messages with Negative as opposed to Positive words were rated as more fun, but only within the context of a Positive message: +M−W (5.00) was higher than +M+W (4.04; *F* = 14.27, *p* < 0.001). When the overall message was Negative, no such difference emerged: −M−W (3.48) did not differ from −M+W (3.29; *F* < 1).

#### Efficient-Wasteful

As with Fun-Serious, all effects were significant, including the main effects of Message [*F*(1, 47) = 83.41, *MSE*  = 3.0, *p* < 0.001] and Word [*F*(1, 47) = 10.37, *MSE*  = 2.1, *p* < 0.01], as well as the interaction [*F*(1, 47) = 11.98, *MSE*  = 1.8, *p* < 0.01]. Once again, cars described in Positive messages (4.75) were rated as more efficient than those in Negative ones (2.48). Unlike the pattern in the Fast-Slow, Exciting-Calm, and Fun-Serious (promotion-based) differentials, however, messages containing Positive words (3.95) were rated as more efficient than those with Negative words (3.28).

All follow-up contrasts to the interaction were significant except one. As with the main effect of Message, car ads with Positive messages were rated as more efficient than corresponding ones with Negative messages: +M+W (5.42) was higher than −M+W (2.48; *F* = 116.34, *p* < 0.001), and +M−W (4.08) was higher than −M−W (2.48; *F* = 34.69, *p* < 0.001). With respect to emotion word effects, and in contrast to the prior differentials, messages with Positive as opposed to Negative words were rated as more efficient, but only within the context of a Positive message: +M+W (5.42) was higher than +M−W (4.08; *F* = 14.27, *p* < 0.001). When the overall message was Negative, no such difference emerged: −M+W (2.48) did not differ from −M−W (2.48; *F* < 1).

#### Comfortable-Uncomfortable

The pattern of results was similar to that for the Efficient-Wasteful differential, with main effects of both Message [*F*(1, 47) = 157.81, *MSE*  = 2.9, *p* < 0.001] and Word [*F*(1, 45) = 81.75, *MSE*  = 1.4, *p* < 0.001], as well as an interaction [*F*(1, 47) = 4.47, *MSE*  = 1.3, *p* < 0.05]. Cars portrayed in Positive messages (5.64) were rated as more comfortable than those in Negative messages (2.54). In addition, messages that used Positive words (4.85) were rated higher than those using Negative words (3.32).

All follow-up contrasts to the interaction were significant. Similar to the direction of the main effect of Message, Positive messages were rated as more comfortable than corresponding Negative ones: +M+W (6.23) was higher than −M+W (3.48; *F* = 143.18, *p* < 0.001), and +M−W (5.04) was higher than −M−W (1.60; *F* = 223.72, *p* < 0.001). With respect to emotion word effects, like Efficient-Wasteful, messages with Positive as opposed to Negative words were rated as more comfortable. Unlike Efficient-Wasteful, however, these effects emerged regardless of message valence: +M+W (6.23) was higher than +M−W (5.04; *F* = 26.70, *p* < 0.001), and −M+W (3.48) was higher than −M−W (1.60; *F* = 66.56, *p* < 0.001). As seen in Figure 2, the interaction arose from the relatively low value within the −M−W condition.

#### Safe-Dangerous

As with Efficient-Wasteful, there were significant main effects of both Message [*F*(1, 47) = 91.98, *MSE*  = 2.7, *p* < 0.001] and Word [*F*(1, 47) = 38.34, *MSE*  = 1.8, *p* < 0.001], as well as an interaction [*F*(1, 47) = 30.08, *MSE*  = 1.6, *p* < 0.001]. Cars described in Positive messages (5.60) were rated as being safer than those in Negative ones (3.33), and messages that used Positive words (5.07) were rated higher than those using Negative words (3.87).

All follow-up contrasts to the interaction were significant except one. In line with the direction of the main effect of Message, Positive messages were rated as safer than corresponding Negative ones: +M+W (5.81) was higher than −M+W (4.33; *F* = 32.09, *p* < 0.001), and +M−W (5.40) was higher than −M−W (2.33; *F* = 137.54, *p* < 0.001). With respect to emotion word effects, messages with Positive as opposed to Negative words were rated as being safer, but only in Negative messages: −M+W (4.33) was higher than −M−W (2.33; *F* = 58.66, *p* < 0.001); for Positive messages, +M+W (5.81) did not differ significantly from +M−W (5.40; *F* = 2.55, *p* = 0.117).

#### Desirable-Undesirable

There was a significant main effect of Message [*F*(1, 47) = 178.84, *MSE*  = 3.0, *p* < 0.001]. Products portrayed in Positive messages were rated as more desirable (5.39) than those in Negative ones (2.05). The main effect of Word was not significant [*F*(1, 47) = 1.32, *MSE*  = 1.9, *p* > 0.25], nor was the interaction [*F*(1, 47) = 1.82, *MSE*  = 1.6, *p* > 0.15].

### Recommendations

**Figure 3:**
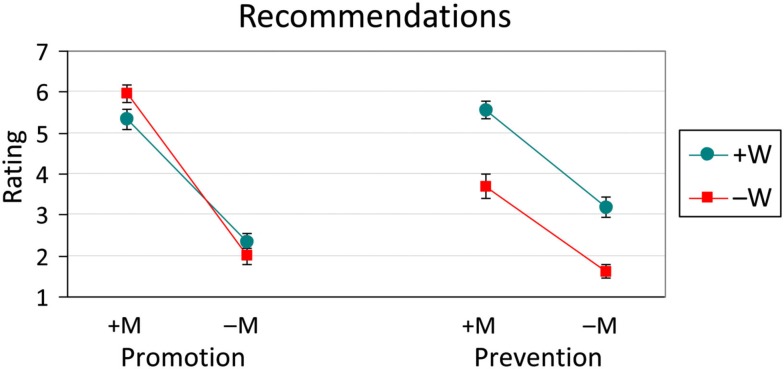
**Product recommendation ratings (with standard error bars) to promotion- and prevention-focused targets across Message (M) × Word (W) conditions**. M and W conditions were either Positive (+) or Negative (−).

The mean promotion- and prevention-focus recommendation ratings (with standard error bars) are presented in Figure 3.

#### Promotion-Focus

The main effect of Message was significant [*F*(1, 47) = 263.20, *MSE*  = 2.2, *p* < 0.001], with Positive messages (5.65) giving rise to higher recommendations than Negative ones (2.15). There was no effect of Word (*F* < 1). The interaction, however, was significant [*F*(1, 47) = 5.08, *MSE*  = 2.2, *p* < 0.05].

Follow-up contrasts to the interaction were significant with one exception. In line with the main effect of Message, Positive messages were more likely to be recommended in a promotion-focus context than corresponding Negative ones: +M+W (5.33) was higher than −M+W (2.31; *F* = 100.94, *p* < 0.001), and +M−W (5.96) was higher than −M−W (1.98; *F* = 175.14, *p* < 0.001). With respect to effects due to the use of emotion words, messages with Negative as opposed to Positive words were more likely to be recommended, but only within the context of a Positive message: +M−W (5.96) was higher than +M+W (5.33; *F* = 4.32, *p* < 0.05). When the overall message was Negative, no such difference emerged: −M−W (1.98) did not differ from −M+W (2.31; *F* = 1.23, *p* > 0.25).

#### Prevention-Focus

There were significant main effects of both Message [*F*(1, 47) = 66.54, *MSE*  = 3.6, *p* < 0.001] and Word [*F*(1, 47) = 49.91, *MSE*  = 2.8, *p* < 0.001], but the interaction was not significant (*F* < 1). Positive messages (4.64) had higher recommendation ratings than Negative ones (2.41), and messages containing Positive words (4.38) had higher ratings than those containing Negative words (2.67).

### Replication study

In order to strengthen the impact of the current results, a replication study was run using a sample equal in size to the original study. Forty-eight members of the University of Aberdeen community (35 female; mean age 20) received course credit for their participation. All key aspects of the experimental method were replicated except that car ads were presented using SuperLab software and reading times were measured via space bar responses from the keyboard.

**Figure 4:**
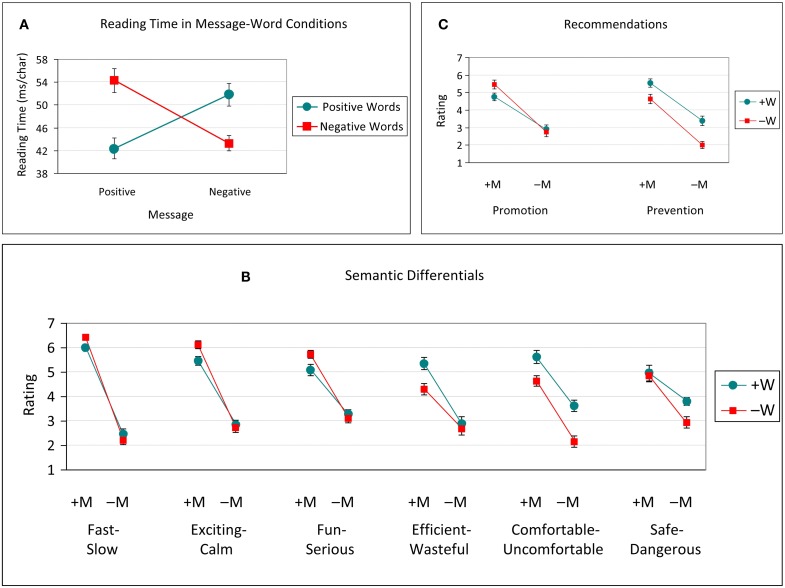
**Replication study averages (with standard error bars) across measures and conditions**. **(A)**, reading time (ms/char) across Message (M) × Word (W) conditions. **(B)**, semantic differential ratings across M × W conditions. M and W conditions were either Positive (+) or Negative (−). Promotion-oriented differentials include Fast-Slow, Exciting-Calm, and Fun-Serious. Prevention-oriented differentials include Efficient-Wasteful, Comfortable-Uncomfortable, and Safe-Dangerous. Each rating scale is 1 to 7, with 7 representing the first word of each pair. **(C)**, product recommendation ratings to promotion- and prevention-focused targets across M × W conditions.

As with the original study, 2 (Message: Positive, Negative) × 2 (Words: Positive, Negative) ANOVAs were performed on the car ad reading times, each of the seven semantic differentials, and the promotion- and prevention-focused recommendations. Condition means (with standard error bars) across all measures are presented in Figure 4 and ANOVA results are presented in Table 2.

**Table 2:** **Analysis of variance results of replication study**.

Measure	Message			Word			Message × Word		
	*F*	*MSE*	*p*	*F*	*MSE*	*p*	*F*	*MSE*	*p*
Reading time	<1			1.61	85	>0.20	62.81	80	<0.001
Fast	405.31	1.8	<0.001	<1			4.29	1.1	<0.05
Exciting	233.01	1.9	<0.001	3.44	0.9	=0.070	5.49	1.4	<0.05
Fun	93.36	2.5	<0.001	1.55	1.8	>0.20	5.77	1.2	<0.05
Efficient	33.66	5.9	<0.001	6.24	2.8	<0.05	3.88	2.4	=0.055
Comfortable	60.51	3.9	<0.001	33.03	2.2	<0.001	<1		
Safe	28.41	4.0	<0.001	3.46	3.3	=0.069	4.43	1.6	<0.05
Desirable	167.61	2.7	<0.001	<1		<1		
Promotion	45.85	5.5	<0.001	2.90	1.2	=0.095	6.23	1.5	<0.05
Prevention	92.18	3.0	<0.001	14.67	4.3	<0.001	1.20	2.1	>0.25

*Main effects of Message and Word and their interaction on measures of reading time, semantic differentials, and recommendations. Degrees of freedom are (1, 47). *MSE*, mean squared error*.

In general, the results of the replication mirrored those of the original study, although the same pattern of significance was not always achieved. The reading time results showed an identical pattern, namely, a significant Message × Word interaction. Reading time was slower when the polarity of Message and Word conditions conflicted (+M−W and −M+W > +M+W and −M−W; all *F*s > 21.85, *p*s < 0.001). For all semantic differentials and recommendations, as before, Positive messages consistently yielded significantly higher ratings than Negative ones. For the Desirable-Undesirable differential, as with the original data, this was the only significant effect. The promotion-focused semantic differentials (Fast-Slow, Exciting-Calm, and Fun-Serious) and the promotion-focused recommendation, however, also showed an additional rating advantage due to Negative words but, in contrast to the original results, this effect tended to be limited to Positive messages (evidenced by significant interactions). The prevention-focused semantic differentials (Efficient-Wasteful, Comfortable-Uncomfortable, and Safe-Dangerous) and the prevention-focused recommendation generally additionally showed, similar to the original results, a significant main effect of Word and as well as a significant Message × Word interaction in which messages containing Positive words typically produced significantly higher ratings.

## Discussion

The current study investigated the role of emotion words in written persuasive messages, specifically, whether such information alone or in interaction with a regulatory perspective helped shape attitudes toward a product. Participants read four car reviews, each of which differed in terms of the valence of the overall message and its constituent words (i.e., +M+W, +M−W, −M+W, and −M−W). Following each review, participants responded to a series of semantic differentials as well as a promotion- and prevention-based recommendation. Analyses were conducted across conditions on reading times and on the semantic differential and recommendation-based ratings. Finally, a replication study was conducted to provide additional support for the current findings.

A congruency effect was observed in reading times – positive reviews were read faster when they contained positive words, and negative reviews were read faster when they contained negative words (see Figures 1 and 4). It is not surprising that additional cognitive resources may be required in processing words that are opposite in valence to the overall message.

More central to the current investigation was the pattern of results across the semantic differential ratings reflecting participants’ attitudes toward the products. The ratings comprised three promotion-oriented differentials (Fast-Slow, Exciting-Calm, and Fun-Serious), three prevention-oriented ones (Efficient-Wasteful, Comfortable-Uncomfortable, and Safe-Dangerous), and one related to the overall impression of the product (Desirable-Undesirable). For all differentials, Positive messages (+M) were rated higher (on the first-mentioned endpoint of each differential, e.g., Fast-, Safe-, etc.), than Negative messages (−M). For desirability, this was the only effect. As can be seen in Figure 2 (see also Figure 4), however, word valence (+W or −W) played a distinguishing role in the remaining differentials. The general pattern showed that, with promotion-oriented differentials, the use of Negative words (−W) gave rise to higher ratings (particularly for Positive messages), while with prevention-oriented differentials, the presence of Positive words (+W) produced an advantage.

A similar set of patterns emerged in the recommendation ratings to promotion- and prevention-focused individuals. For both types of ratings, Positive messages (+M) produced higher recommendations than Negative messages (−M). As with the promotion- and prevention-oriented semantic differentials, word valence (+W or −W) similarly modulated promotion- and prevention-focused recommendations (see Figures 3 and 4). Recommendations to a promotion-focused individual were higher after reading Positive messages (+M) when that message contained Negative words (−W); there was no difference in ratings related to word valence after reading Negative messages. Recommendations to a prevention-focused individual, in contrast, were higher after reading either Positive or Negative messages (+M or −M) when those messages used Positive words (+W). This differs from participants’ ratings of how desirable they, themselves, judged each product to be, where only message and not word valence played a role. Regulatory focus was therefore seemingly applied retrospectively as participants judged the suitability of a car for someone else, an individual with either an explicit promotion- or prevention-focus.

Thus, the pattern of effects differed between the initial on-line processing of product information (reading time) and the later measures that reflected the formation of attitudes toward each product (semantic differential and recommendation ratings). That is, on-line reading time only demonstrated congruency effects of Message and Word valence (+M+W and −M−W were read faster than +M−W and −M+W). While the semantic differential and recommendation ratings were always higher for Positive than for Negative messages, word valence played a key role in discriminating between promotion and prevention regulatory focus. For promotion-oriented differentials and promotion-focused recommendations, Negative words, particularly when in a Positive message, led to higher ratings (+M−W was higher than +M+W, while −M−W was only sometimes higher than −M+W). For prevention-oriented differentials and prevention-focused recommendations, Positive words in most cases gave rise to higher ratings irrespective of message type (+W was higher than −W).

A key focus of this study was to determine how the use of emotion words in a persuasive message affected attitude formation. Although participants were not induced into a specific motivational framework prior to reading the product reviews, both sets of product ratings (semantic differentials and recommendations) implicitly encouraged participants to adopt promotion- and prevention-focused perspectives. Our findings demonstrated that word valence did influence attitudes. While such effects were smaller in magnitude than those due to message valence, the effects were differentially expressed within promotion- and prevention-focused contexts. Regardless of message valence, Negative words notably served to enhance promotion-based ratings. In contrast, word valence in prevention-based ratings served to intensify the difference between Positive and Negative messages – whether they enhanced Positive messages, further demoted Negative ones, or both is not clear.

One explanation for these findings may be in how word valence is differentially interpreted depending on the regulatory context. For promotion-based attitudes, Negative words were not taken at “face value,” particularly in Positive messages. For prevention-based attitudes, negative as well as positive information – either in the form of the message or its component words – acted cumulatively. A promotion-focused judgment enables more ambiguity or versatility in interpreting the emotional meaning of words (i.e., “wicked” can be exciting), whereas a prevention-focused judgment relies on the literal emotional meaning (i.e., “wicked” is just evil). It has been demonstrated that processing styles can be modulated by the regulatory focus of the actor in complex ways. For example, promotion-focus has been associated with more abstract processing and possibly more creative activity (e.g., Semin et al., 2005; Vaughn et al., 2008; but cf. Baas et al., 2008). It therefore seems plausible to suggest that incongruities between discourse and word levels would be dealt with more flexibly by those in a promotion context and that this would lead to different product ratings.

The issue of regulatory fit – defined as a match between regulatory focus of the individual and the promotion- or prevention-appeal of the message, leading to greater ease of message processing and a transfer of value to the target (Freitas and Higgins, 2002; Cesario et al., 2008; Mourali and Pons, 2009) – has also been shown to be relevant to persuasion. In the current study, however, both types of positive reviews (+M+W and +M−W) and both types of negative reviews (−M+W and −M−W) were rated as equally desirable and undesirable, respectively, indicating no differences in ease of processing across conditions.

While our study may only provide a provisional account of the role that emotional language plays in persuasive messages, it nonetheless represents an initial exploration of these issues. For example, our explanation of regulatory focus effects was somewhat constrained by our procedures. Regulatory focus was manipulated implicitly in the nature of the semantic differentials used and vicariously through product recommendations to specific individuals. There are potential drawbacks to such procedures in comparison to traditional regulatory focus manipulations. First, the implicit or vicarious nature of the task should serve to weaken focus effects. Second, since the ratings were performed after reading each review, they were memory-based and could be influenced by the other ratings of the product. Nevertheless, our results did demonstrate distinctive effects of discourse and word-level manipulations. It would be interesting to determine whether such effects generalize to other products or markets.

In summary, while products were consistently rated higher when depicted in Positive as opposed to Negative messages, the effects due to Positive and Negative words were more complex. In promotion-based ratings, Negative rather than Positive words gave rise to higher ratings. In prevention-based ratings, Positive and Negative words served to raise and lower ratings, respectively, independent of the message. These findings point to a fundamental difference in the style of processing with respect to promotion- and prevention-focused aspects of products and to the influence of emotional language in shaping attitudes.

## Conflict of Interest Statement

The authors declare that the research was conducted in the absence of any commercial or financial relationships that could be construed as a potential conflict of interest.

## Appendix

### Car reviews

#### Positive message, positive words

The Ford Kiss is a relatively new addition to the £12,000–15,000 hatchback class and at first glance measures up very favorably to its established peers. The 1.5-l six valve engine packs in a monumental 140 bhp, sprints from 0 to 60 in just 5 s and delivers an astonishing top speed of 158 mph. While this machine’s awesome pace is most evidently on display on motorways and A-roads, it is complimented by pristine handling and phenomenal grip which ensures supreme confidence when cornering hard at any speed. This combination of power and finesse ensures sparkling performance in all driving environments.

This classy vehicle also comes lavishly equipped with an extensive range of features designed to make the interior as luxurious as possible. Immaculately upholstered and surprisingly supportive seats make it clear that comfort has priority, while there is abundant useable room inside the cabin, as well as a large boot.

In addition to the quality feel there are practical applications too. The Kiss is very economical to run and service, making it great value. Finally a generous range of impressive safety features such as state-of-the-art alarms, immobilizer, and smart-key entry system ensure that you sleep soundly in your bed knowing that your car is protecting itself.

#### Positive message, negative words

The 3-door Daewoo Scorpion possesses blistering pace for such a hefty car with an intimidating top speed of 160 mph, and the basic model’s 1.5 l, six valve engine delivering a screeching 155 bhp. When setting off this car will emit a scream to chill the spine as it climbs to 60 mph in only 8 s. While cruising the growl of the engine is not too loud, but provides an ever-present reminder of the wild beast lurking under the bonnet. There’s more to this machine than its ferocious speed, with razor-sharp handling allowing you to attack the tightest corners with venom, and affording you the confidence to thunder through town centers and snake along twisty B-roads.

This car punches above its weight in the 12,000–15,000 pound small hatchback class. It is lighting fast, bulkier, and less pricey than the majority of its rivals. As well as its stirring performance, the interior is cavernous and the monstrous boot capable of greedily devouring a set of golf clubs. All this will keep you calm during the daily hustle and grind of the city.

The Scorpion employs a number of passive safety features to prevent nasty accidents and injuries. These include airbags, front and side crumple zones, and skeletal front pillars to reduce blind spots. As well as the expected alarm system the vehicle comes equipped with dead-locks which should help to discourage thieves and vandals.

#### Negative message, positive words

The newly released Fiat Casino is a compact hatchback in a comparable style to the popular Renault Clio. Like its French cousin it has a 1.2-l engine and three doors, but that’s where the similarity ends. With a sedate top speed of 105 mph, and the ability to stroll from 0 to 60 in a colossal 10 s, all you will be able to do is sit back, relax, and watch as you are overtaken by better, more advanced cars, and perhaps also a child on a bicycle. Handling is neither superb nor exciting, with the expected nimbleness absent, and new suspension offering the poise and grace of rollercoaster cart.

The Casino is far too serene for a sports or muscle car, but we suspect it is rather too extravagantly complicated to appeal to sensible housewives either. While an amazing array of dazzling techno-gadgets are at your fingertips you would need an advanced degree to enjoy them.

At a standard £14,500 this car is not easy on the wallet, and liberal fuel consumption means you will soon have to part with more of your cash at the pumps. Add to this the less than first-class performance and an interior which looks comfortable and classy but is made of polyester and you would do well to invest your money elsewhere.

#### Negative message, negative words

The three door Nissan Storm has a 1.4-l engine which delivers a top speed of 122 mph. Unfortunately it offers only 105 bhp meaning that acceleration is sluggish and you will inevitably be left plodding along, seething with jealousy as less inferior cars leave you trailing in their wake. And it is not just the performance which spoils this car. Control is awkward, particularly at high speeds, and a tortured clanking under the bonnet betrays sloppy engineering which threatens to turn into more critical problems sooner rather than later. The all round ride and handling leave you feeling dissatisfied and unfulfilled.

The Storm is marketed as being compact, but the interior is cramped and brick-like suspension means the worst bumps can be felt as bone-shaking shudders. There is little storage room, with a miniscule glove-compartment and shallow boot limiting load-carrying ability. Also absent is sufficient headroom, with a low roofline reducing comfort and hampering rear visibility.

Other strange omissions include many of the safety features which tempted buyers of the previous model, leaving this particular vehicle vulnerable to thieves. Combine this with a thirsty engine and disappointing performance and what you have is a costly vehicle which compares poorly to other cars in its class.
